# Supplementary material for: Flying high on low cost: Success in the low-cost airline industry
Source: PLoS One. 2023 Dec 21;18(12):e0294638. doi: 10.1371/journal.pone.0294638 (PMC10734975; doi:10.1371/journal.pone.0294638)
Supplement: S2 Table — (DOCX) [file pone.0294638.s002.docx]

**S2 Table.** **Parsimonious solution (success)**

|  | raw coverage | unique coverage | consistency |
| --- | --- | --- | --- |
| Services*~On-time | 0.429 | 0.214 | 1 |
| Size*~On-time | 0.357 | 0 | 1 |
| Size*~Services | 0.286 | 0 | 1 |
| ~Services*On-time | 0.429 | 0 | 1 |
| Size*Productivity | 0.286 | 0 | 1 |
| On-time*Productivity | 0.429 | 0 | 1 |
| solution coverage: 1 |  |  |  |
| solution consistency: 1 |  |  |  |
