# Supplementary material for: Flying high on low cost: Success in the low-cost airline industry
Source: PLoS One. 2023 Dec 21;18(12):e0294638. doi: 10.1371/journal.pone.0294638 (PMC10734975; doi:10.1371/journal.pone.0294638)
Supplement: S3 Table — (DOCX) [file pone.0294638.s003.docx]

**S3 Table. Complex solution (~success)**

|  | raw coverage | unique coverage | consistency |
| --- | --- | --- | --- |
| Group*Services*On-time*~Productivity*~Long-haul | 0.429 | 0.143 | 1 |
| ~Size*Group*Services*On-time*~Productivity | 0.429 | 0.143 | 1 |
| ~Size*~Group*~Services*~On-time*~Productivity* ~Long-haul | 0.143 | 0.143 | 1 |
| ~Size*Group*~Services*~On-time*Productivity* ~Long-haul | 0.143 | 0.143 | 1 |
| ~Size*~Group*~Services*~On-time*Productivity* Long-haul | 0.143 | 0.143 | 1 |
| solution coverage: 1 |  |  |  |
| solution consistency: 1 |  |  |  |
