# Supplementary material for: Flying high on low cost: Success in the low-cost airline industry
Source: PLoS One. 2023 Dec 21;18(12):e0294638. doi: 10.1371/journal.pone.0294638 (PMC10734975; doi:10.1371/journal.pone.0294638)
Supplement: Attachment — Submitted filename: Response letter 2.docx [file pone.0294638.s006.docx]

Dear editor,

We thank you and both reviewers for the feedback and the opportunity to review the paper. While we sometimes argue against some of the reviewers' comments, we considered them thoroughly, and we are much thankful for them. All of them were relevant, and many potential readers of our manuscript would raise the same concerns. In response to these comments, we clarified numerous things as well as provided a more detailed account of our work. Below we respond to the reviewers’ comments:

**Reviewer #1**

1. The authors adopt the QCA investigative approach and state that they use crisp set QCA. While QCA itself seems a good choice (given the number of cases being investigated) I would like to have seen rationale for the choice of csQCA over fuzzy set fsQCA. The authors point to the use of fsQCA 3.0 software (which support fuzzy sets QCA) in the Methodology section. Are the methods so similar that an analysis tool built for fsQCA can be immediately applied to csQCA formulation?

Yes, the methods are similar in the sense and fsQCA is more complex variant of csQCA (abstracting away from some differences). The fsQCA software is not built only for fsQCA – csQCA functionality is its basis. The software was co-developed by Charles Ragin, the “founding father” of QCA analysis (that started with csQCA).

We choose csQCA for its clarity of interpretation as well as suitability for our research. The major disadvantage of csQCA is that it uses binary values of conditions (either present or absent) – making it sensitive to cut-off levels. However, it is important to note that fsQCA also works with calibration/cut-off levels – it only has more of them. That makes it less sensitive (e.g., the difference between 0.33 and 0.66 is smaller than between 0 and 1 – even though in QCA the differences do not work as mere subtractions) but also creates an additional burden for justifying calibration criteria and for interpretation (in csQCA it is much easier for a reader to go back to cases and see what airlines represent individual combinations of conditions). In the end, it is a trade-off without a clear choice. In our case, we believe that parsimony (csQCA) brings more value.

2. The authors devise 6 conditions and a success attribute which they then use in generating causality rules. Each condition, and the success label, have multiple indicators and calibration criteria. These are used in generating the truth table for each airline. IT is not made clear how the indicators and calibration criteria are combined to determine a binary 0/1 value for the condition for each airline. For instance, is it the case that satisfying a single indicator/calibration criteria is sufficient for the airline to be rated a 1 for the condition?

This was indeed not written in the paper; thank you for raising this issue. We explained in the manuscript that most indicators needed to be fulfilled to mark the condition as present.

3. I would like to have seen a discussion on candidate conditions that were considered but rejected, the rationale for such decisions, what POA said about the conditions,...

There is a short discussion on this topic in the section “Business model attribues” that is now slightly extended. In general, we tried to avoid rejecting any general attributes of business models – see the argument for that in response to comment 2 of Reviewer 4.

4. I would like to have seen some analysis of the conditions that showed they are not correlated.

Thank you very much for raising this concern. While correlation between independent variables is critical in correlational methods (such as regression), it does not create the same issue in configurational methods. The simple explanation is that in correlational methods, the math behind estimates the effects of individual variables separately from the others (so multicollinearity would make it difficult/impossible). In contrast, in configurational methods, we are interested how conditions (variables) affect the outcome together. That means if we had perfectly correlated variables, they would both appear or not appear simultaneously in reported configurations. Mathematically, they do not cause any problems.

On the other hand, such a simultaneous appearance of conditions could be problematic for interpretation. Luckily, since QCA relies on extensive theoretical and contextual understanding of a studied problem, one may be able to clearly explain what is happening in the study's findings. If this is not possible, the two conditions should be either merged or one of them abandoned. We believe that we can justify the role (and added value) of each of the conditions used in our study.

The only strong correlation (if we abstract from the limitation of having binary values) across conditions is between Services and Productivity. We deal with it thoroughly in interpreting our findings/discussion. We believe that abstracting the negative relationship between the two conditions would harm the clarity of interpretation of our findings, and we are both theoretically and contextually able to justify the use of both conditions.

5. The authors make note of the Long Haul condition and the are hesitant to interpret its role in determining success of LCC. Further work needs to be done to include non-European LLCs that do service a wide geographical area - for instance Scoot, Singapore Airline's LCC.

Thank you for raising this concern, which we certainly omitted in our study's limitations. Regional airline markets are still considerably different even after significant deregulation. Our study relies on European LCCs operating in a different market than the airlines you mentioned. Extending our study to non-European LCCs would require not only adding a few airlines but likely multiply our set of cases (to have it balanced geographically). This would be problematic for two reasons: 1) Unavailability of data (even for European airlines, it was problematic/impossible to collect all the data we needed) and 2) Confusion in findings. When we acknowledge that regional markets differ (which we believe, and we also see it in your comment), then mixing them together would only make some successful business models less visible (though it may make clear what stands out across all the markets – however, this would be much simpler picture raising concern of added value). In the end, we believe it would make the most sense to analyze such a set of cases in separate analyses. That would mean we would again only have European LCCs – and, besides them, other regional markets. This study would be interesting but certainly exceeds a format of a stand-alone paper (note the information would necessarily multiply – which makes it more suitable for a follow-up study, in case 1) data is available).

6. Some minor comments (typos, etc.)

Fixed – thank you very much for your careful reading of the text!

Reviewer #2

**1. Compared to previous research, the contribution of this study needs to be adequately specified. The authors can highlight the significance and uniqueness of this study compared to existing literature, such as:**

**• Loureiro, S. (2016). In-flight attributes and mindful passengers: Qualitative comparative analysis (QCA) of relationship quality and behavioral intentions configurations. In-flight attributes and mindful passengers: Qualitative comparative analysis (QCA) of relationship quality and behavioral intentions configurations, 1032-1033.**

**• Hvass, K. A. (2012). A Boolean Approach to Airline Business Model Innovation.**

• Ott, U. F., Sinkovics, R. R., & Hoque, S. F. (2018). Advances in qualitative comparative analysis (QCA): Application of fuzzy set in business and management research. C. Cassell, A. Cunliffe, & G. Grandy, The SAGE Handbook of Qualitative Business and Management Research Methods: Methods and Challenges, 414-430.

Thank you very much for pointing us to these references. There is indeed a couple of studies that used QCA in the airline industry but none directly comparable to ours. However, after careful consideration, we integrated Hvass (2012), who is likely the closest to what we do in our research, with his focus on airlines' business models (though much more narrow on configurations of innovative activities, not business models in general).

2. The literature review should examine the relationship between the independent variables utilized in the study and their correlation with the success criteria in Table 2.

We differentiate our study from previous correlational studies – we refer to the first part of our discussion. While we see the merits of these correlational studies, we believe they do not bring us the insights we would need to conduct our study (i.e., how business model attributes TOGETHER contribute to success or its absence). In this regard, we believe that our study is rather exploratory, and we had no expectations of configurations that would lead to success or its absence. In this regard, note the missing hypotheses.

Based on this, we focused on general defining attributes of business models (unless they do not distinguish between individual airlines – see our response to your comment 4) – and explored whether and what configuration will stand out. Given that we are looking at configurations, focusing on attributes previously found to be related to success (or its absence) would likely lead to missing variable bias.

We added a short note on this aspect to the manuscript as we believe our approach was not previously clear and thus confusing. Thank you for highlighting this issue.

3. Please provide a more detailed explanation of the method's development and the software employed in this study. Additionally, clarify the reason for selecting FsQCA 3.0 as the chosen software over alternatives such as nvivo, Atlas.ti, or other similar tools.?

Regarding the first part of this comment (method development), we refer back to replies to comments 1-3 (Reviewer 3) and 2 (Reviewer 4). We also reply on the use of fsQCA 3.0 software there partially, but to further distinguish from the mentioned software: NVivo and Atlas.ti are tools used primarily for qualitative data analysis. They do not provide the depth of tools needed for QCA (although some basic analysis is possible) – fsQCA is the standard software used by the QCA community. Other relevant alternatives are, for example, QCA packages for R, etc. However, given our use of csQCA and not any of the more advanced QCA variants, there is no difference between these standard QCA software solutions.

We also hope that using fsQCA software as an illustration in the chapter you referred to in the first comment (Ott, Sinkovics and Hogue, 2018) further supports our claims.

4. In lines 163-168, How can the authors ensure that paid onboard refreshments, the use of secondary airports, tight seat pitches, and seat widths are not determining factors in differentiating between successful and unsuccessful ventures? Are there any references supporting this claim?

In this regard, we rely on general defining features of LCC business models (see Whyte and Lohmann, 2020; Williams, 2001; O’Connel and Williams, 2005). When all (or virtually all) the cases-airline share the attribute it is not possible to use it in analysis – though they may represent a necessary condition for success. In this regard, Necessary Condition Analysis and more detailed data (note our use of csQCA) may provide interesting insights. We already pointed to this suggestion in the discussion and elaborated this part in the new version.

5. How was the quantitative value in calibration criteria Table 1 defined or derived? Are these numbers correlated with airline success in previous studies?

As implied in our response to comment 2 (Reviewer 4), we did not rely on correlational studies. Instead – as was briefly and now more extensively covered in the manuscript – we relied on values that assign airlines into two groups or on our knowledge of the industry and individual airlines. We acknowledge this is the most qualitative aspect of our study – and should be taken as such, including its limitations.

6. How were the success calibration criteria in Table 2 defined? For instance, what is the reason behind setting the cost per available seat kilometer to less than 4 U.S. cents? Is there a specific reason for selecting 3.5 points as the calibration value in Customer reviews??

See above.

7. Have the authors conducted a sensitivity analysis on the selection of the independent variable calibration (IV) and outcome calibration (DV) to ensure the robustness of the results??

Yes, we conducted multiple tests focused especially on the calibration criteria (but also on other aspects of the analysis). The findings are relatively stable – when we acknowledge the general sensitivity of csQCA to calibration criteria. In this regard, we believe that we can build on the insight into individual cases that allows us to build on a more qualitative assessment of the situation of individual airlines.

8. The interpretation of the results could be more precise. The authors should explicitly mention the specific result or table to which they are referring in their explanations.

We connected the interpretation much more to both specific tables or to the conditions we are referring to.

The results part has been extended in general to provide a more in-depth description of our findings.

9. Please establish connections between your findings and previous studies. Additionally, provide plausible explanations for the outcomes in the discussion.

We extended our section ‘Interpretation of results’, which serves the role of providing explanations that would normally appear in the discussion. However, given the expected unfamiliarity of most readers with the QCA method, we believe that ending the results section with both intermediate solutions would make it much harder for readers to 'digest' what we found out. Thus, we would like to keep the 'Interpretation of results' as a bridge to a much more general discussion in the paper.

Once again, thank you for your feedback. It considerably helped us in improving our manuscript.

Yours sincerely, the authors
